# Climate-Resilient Crops: Integrating AI, Multi-Omics, and Advanced Phenotyping to Address Global Agricultural and Societal Challenges

**DOI:** 10.3390/plants14172699

**Published:** 2025-08-29

**Authors:** Doni Thingujam, Sandeep Gouli, Sachin Promodh Cooray, Katie Busch Chandran, Seth Bradley Givens, Renganathan Vellaichamy Gandhimeyyan, Zhengzhi Tan, Yiqing Wang, Keerthi Patam, Sydney A. Greer, Ranju Acharya, David Octor Moseley, Nesma Osman, Xin Zhang, Megan E. Brooker, Mary Love Tagert, Mark J. Schafer, Changyoon Jeong, Kevin Flynn Hoffseth, Raju Bheemanahalli, J. Michael Wyss, Nuwan Kumara Wijewardane, Jong Hyun Ham, M. Shahid Mukhtar

**Affiliations:** 1 Department of Biological Sciences, Clemson University, 132 Long Hall, Clemson, SC 29634, USA; dthingu@clemson.edu; 2 Department of Plant Pathology and Crop Physiology, Louisiana State University Agricultural Center, Baton Rouge, LA 70803, USA; sgouli@agcenter.lsu.edu (S.G.); jham@agcenter.lsu.edu (J.H.H.); 3 Department of Agricultural & Biological Engineering, Mississippi State University, Starkville, MS 39762, USA; tcc337@msstate.edu (S.P.C.); sbg195@msstate.edu (S.B.G.); xzhang@abe.msstate.edu (X.Z.); mltagert@abe.msstate.edu (M.L.T.); nuwanw@abe.msstate.edu (N.K.W.); 4 Center for Community Outreach Development, University of Alabama at Birmingham, Birmingham, AL 35294, USA; kabusch@uab.edu; 5 Department of Plant and Soil Sciences, Mississippi State University, Starkville, MS 39759, USA; rv385@msstate.edu (R.V.G.); rajubr@pss.msstate.edu (R.B.); 6 Department of Genetics & Biochemistry, Clemson University, 105 Collings St., Biosystems Research Complex, Clemson, SC 29634, USA; tzhengz@clemson.edu (Z.T.); yiqingw@clemson.edu (Y.W.); kpatam@clemson.edu (K.P.); sagreer@clemson.edu (S.A.G.); 7 Department of Agricultural Economics and Agribusiness, Louisiana State University Agricultural Center, Baton Rouge, LA 70803, USA; rachar3@lsu.edu (R.A.); mschafer@agcenter.lsu.edu (M.J.S.); 8 Dean Lee Research and Extension Center, Louisiana State University Agricultural Center, Alexandria, LA 71302, USA; dmoseley@agcenter.lsu.edu; 9 Department of Agricultural Education, Leadership, and Communications, Mississippi State University, Starkville, MS 39762, USA; noo10@msstate.edu; 10 Rural Sociology and Agricultural Extension Department, Faculty of Agriculture, Cairo University, Al Giza 12613, Egypt; 11 Department of Sociology, University of Alabama at Birmingham, Birmingham, AL 35233, USA; brookerm@uab.edu; 12 Red River Research Station, Louisiana State University Agricultural Center, Bossier City, LA 71105, USA; cjeong@agcenter.lsu.edu; 13 Department of Biological and Agricultural Engineering, Louisiana State University Agricultural Center, Baton Rouge, LA 70803, USA; khoffseth@agcenter.lsu.edu; 14 Department of Cell, Developmental, and Integrative Biology, University of Alabama at Birmingham, Birmingham, AL 35233, USA; jmwyss@uab.edu

**Keywords:** climate change, abiotic stress, machine learning, microbiome, epigenomics, genomics, proteomics, metabolomics

## Abstract

Drought and excess ambient temperature intensify abiotic and biotic stresses on agriculture, threatening food security and economic stability. The development of climate-resilient crops is crucial for sustainable, efficient farming. This review highlights the role of multi-omics encompassing genomics, transcriptomics, proteomics, metabolomics, and epigenomics in identifying genetic pathways for stress resilience. Advanced phenomics, using drones and hyperspectral imaging, can accelerate breeding programs by enabling high-throughput trait monitoring. Artificial intelligence (AI) and machine learning (ML) enhance these efforts by analyzing large-scale omics and phenotypic data, predicting stress tolerance traits, and optimizing breeding strategies. Additionally, plant-associated microbiomes contribute to stress tolerance and soil health through bioinoculants and synthetic microbial communities. Beyond agriculture, these advancements have broad societal, economic, and educational impacts. Climate-resilient crops can enhance food security, reduce hunger, and support vulnerable regions. AI-driven tools and precision agriculture empower farmers, improving livelihoods and equitable technology access. Educating teachers, students, and future generations fosters awareness and equips them to address climate challenges. Economically, these innovations reduce financial risks, stabilize markets, and promote long-term agricultural sustainability. These cutting-edge approaches can transform agriculture by integrating AI, multi-omics, and advanced phenotyping, ensuring a resilient and sustainable global food system amid climate change.

## 1. Introduction

The accelerating impacts of climate change are profoundly altering agricultural landscapes worldwide, posing serious threats to food security, economic stability, and societal well-being [1]. Unpredictable weather patterns, including prolonged droughts, intense heat waves, devastating floods, and the proliferation of novel pests and diseases, are increasingly disrupting traditional farming practices and reducing crop yields [2]. These challenges are increased by the growing global population requiring more food, thus placing immense pressure on existing agricultural systems [3,4]. Agriculture is inherently dependent on stable environmental conditions and thus is one of the most vulnerable sectors to the multifaceted impacts of climate change [5]. Rising global temperatures are already leading to heat stress in many crops, disrupting vital physiological processes such as photosynthesis and pollination, and ultimately reducing yields [6]. Changes in precipitation patterns, characterized by increased frequency and intensity of droughts in some regions and floods in others, create water scarcity or waterlogged soils, both detrimental to plant growth and survival [7]. Furthermore, altered temperature and humidity conditions are facilitating the geographic expansion and increased virulence of agricultural pests and diseases, demanding new management strategies [8]. The increased frequency of extreme weather events, such as hurricanes, cyclones, and hailstorms, can cause catastrophic damage to crop and agricultural infrastructure, leading to significant economic losses and food shortages [9]. These climate-induced stresses adversely affect not only crop yields but also soil health, water resources, and the long-term sustainability of agricultural systems, presenting a multifaceted challenge for both farmers and policymakers.

Addressing these growing challenges, the development and widespread adoption of climate-resilient crops have become a critical global priority. Developing crops that possess enhanced tolerance or resistance to various abiotic and biotic stresses linked to climate change is crucial for ensuring future food security, protecting the livelihoods of vulnerable populations, and advancing a more sustainable and equitable agricultural future [10]. Climate resilience encompasses a range of desirable traits, including the ability to maintain yield stability under fluctuating environmental conditions, efficient resource utilization, and enhanced nutritional quality [11]. The quest for climate-resilient crops has been significantly accelerated by the integration of several powerful and rapidly evolving technologies. Artificial intelligence (AI) is playing an increasingly vital role in analyzing vast datasets from genomics, phenomics, and environmental monitoring to identify complex relationships and predict optimal breeding strategies for resilience [12]. AI algorithms can assist in identifying superior germplasm, optimizing resource management, and developing predictive models for pest and disease outbreaks under changing climate scenarios [13]. The analytical capabilities of AI complementing multi-omics approaches offer an integrated view of the molecular mechanisms that govern plant responses to environmental stresses [14]. Simultaneous analysis of these different biological layers can identify key genes, proteins, and metabolic pathways associated with resilience traits, enabling more targeted breeding efforts [15,16,17]. Nevertheless, advanced phenotyping technologies use high-throughput platforms with sensors, imaging, and automation to non-destructively measure complex plant traits across scales. When combined with genomic and environmental data, they provide insights into genotype–phenotype relationships, facilitating the selection of climate-resilient crop varieties [18,19]. This review emphasizes the key technological advancements, specifically AI, multi-omics, and advanced phenotyping, and how synergistic integration of these tools holds immense promise for accelerating our ability to cultivate crops fit for a changing world, thereby addressing critical agricultural and societal challenges.

## 2. Climate Change and Agricultural Stress

### 2.1. Abiotic Stress Factors

#### 2.1.1. Drought and Water Scarcity

Water scarcity, exacerbated by global warming and human activities, poses a significant threat to ecosystems and human survival. Uneven precipitation patterns, predicted to worsen with increased Arctic stream flow and decreased subtropical rainfall, are driving hydrological and agricultural droughts [20]. According to the United Nations Convention to Combat Desertification [21] report, 44% of droughts have occurred on the African continent, with over 300 drought events recorded in the last 100 years, including 134 events between 2000 and 2019. Over the last century, Europe has experienced 45 drought events, affected millions of people, and resulted in USD 27.8 billion in losses [22]. In terms of population, more than 2 billion people were affected by droughts in the 20th century, mainly in North Africa, the Middle East, and Australia [23]. These changes affect billions worldwide and pose a significant threat to food security and socioeconomic stability. Projections indicate a substantial expansion of arid regions, potentially displacing over 700 million people by 2030 [24]. Global freshwater withdrawals reached 3.9 trillion m^3^ in 2017, up from 500 billion m^3^ in 1900, largely due to population growth and resource-intensive consumption patterns [25]. Freshwater demand for food production is expected to double by 2050, supporting a population of 10 billion people, but its availability remains uncertain due to rising concerns about water scarcity and socio-economic impacts [26]. Additionally, emissions of CO_2_ and methane have increased global temperatures by 0.85 °C from 1880 to 2012, with projections exceeding 1.5 °C this century [20].

Drought poses a significant threat to animals and crops, affecting up to 45% of the world’s agricultural land, which is home to 38% of the global population [21]. In the US, drought is increasingly common, currently costing an average of USD 9.6 B per year, and it is thus considered one of the most critical economic problems and natural threats to the planet. Drought, combined with heat stress, can severely impact agriculture, resulting in estimated losses of USD 200 billion in the USA between 1980 and 2012 [27]. Plants are vulnerable to moisture stress, particularly due to drought caused by limited water supply or high transpiration rates [28]. Prolonged drought leads to declining plant fitness and yield [29,30,31]. Major crops face significant yield risks due to water scarcity, with projected losses of 7 to 8.1% for soybean, 9 to 12% for wheat, and 18.1 to 19.4% for rice by the end of the 21st century [9,32,33].

**Figure 1:**
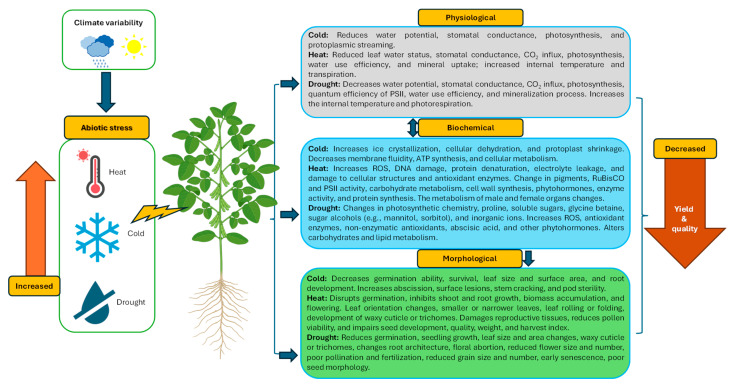
Schematic diagram of effect of heat, cold, and drought stress on crop plants.

In semi-arid and drought-prone areas, the co-occurrence of drought and heat stress has a detrimental impact on plants [34,35]. Heino et al. (2023) noted that heat stress often coincides with drought worldwide during the reproductive stages of wheat, rice, and maize [36]. This combined stress affects gas exchange, photosynthetic pigments, enzyme activities, and biochemistry [37] (Figure 1). Furthermore, concomitant drought and heat waves during reproductive stages cause abnormal anther development, reduced pollen viability, ovule abortion, and inhibited flowering [38,39]. In maize, the co-occurrence of drought and heat stress increases ROS and malondialdehyde levels, leading to reduced nutrient uptake and diminished growth [40]. Similarly, soybeans experience a 7% reduction in seed weight due to the interactive effects of drought and heat during flowering [34].

#### 2.1.2. Extreme Temperature Stress

Temperature significantly impacts organisms’ distribution across latitudinal and altitudinal gradients [41]. Plant life exists within a limited temperature range, from −10 °C [e.g., temperate woody trees] to a specific threshold for arid cacti. Each organism has an optimal temperature range that maximizes growth and function, while deviations can halt biological processes, leading to stress or death depending on intensity and duration [42]. Low or non-freezing temperatures (0–15 °C) can induce substantial injury and mortality in tropical and subtropical plants [43]. Tropical and subtropical species, such as maize, soybeans, cotton, tomatoes, and bananas, exhibit rapid injury symptoms when exposed to temperatures below 10–15 °C, due to their limited adaptation to temperate climates [44] (Figure 1). Cold stress disrupts plant growth and development. The severity of cold stress effects is modulated by plant cellular morphology, biochemistry, physiological functions, and developmental stage [45]. Symptom manifestation varies across plant species and tissues, reflecting differential sensitivities [46]. In rice, early-stage cold stress can have lasting impacts on later growth, leading to cellular membrane damage and growth retardation [47]. Visual symptoms of cold injury include tissue damage, surface lesions, stem cracking, metabolite leakage, leaf wilting, chlorosis, necrosis, leaf curling, and discoloration [48,49].

Temperature stress influences photosynthesis, nutrient cycling, protein synthesis, and phytohormone regulation [50]. Future projections indicate that heatwaves and extreme temperatures will become more frequent and intense, with the prediction of a temperature increase of 2 to 3 °C over the next 30 to 50 years [20]. Even with a 1 °C increase, prolonged heat waves can result in up to 38% yield loss in wheat [51,52]. Global average temperatures have risen by 0.8 °C since 1990, with nighttime temperatures rising 1.4 times faster than daytime temperatures [53]. This poses a significant threat to agriculture, resulting in reduced grain yields of up to 6% in wheat (*Triticum aestivum* L.), 3.2% in rice (*Oryza sativa*), 3.4% in maize (*Zea mays*) and 2.8% in soybean for every 1 °C rise in nighttime temperature from their control [54,55,56]. Heat stress particularly impacts reproductive stages in crops, reducing pollen viability and seed set [57,58]. For example, heat stress can disrupt both male and female reproductive organs, leading to significant yield losses [58]. Moreover, the effect of heat waves often coincides with high vapor pressure deficits (VPD), beyond a certain point, higher temperature with high VPD adversely affect the pollen development, thus reducing sexual reproduction and grain yield formation [59,60] [Figure 1]. Heat stress occurring for 2 to 5 days around flowering negatively impacts reproductive organ function and seed set in rice [61,62], affects the anthesis-silking interval in maize [63], and decreases pollen shedding in wheat [52,64], leading to irreversible yield loss through reduced floret fertility and seed number [38,65,66].

### 2.2. Agricultural Management Strategies and Abiotic Stresses

Effective agricultural and water management strategies are essential for mitigating abiotic stresses such as drought, waterlogging, and salinity [67,68,69]. By enhancing water use efficiency and integrating conservation practices, farmers can ensure crops receive adequate moisture, minimize water loss, and strengthen resilience to environmental challenges [70]. Employing soil amendments, cover crop practices, and sound soil management, along with appropriate crop selection, enables the maintenance of productivity under changing climatic conditions [71,72,73]. The application of soil amendments, including thermally and hydrothermally processed materials such as biochar and hydrochar, represents an innovative approach to enhance soil health and mitigate abiotic stresses like drought, salinity, and temperature extremes [74,75,76]. Biochar, produced via pyrolysis under high temperatures and limited oxygen, and hydrochar, generated through hydrothermal carbonization (HTC) under high pressure and temperature in the presence of water, are stable, carbon-rich products. These materials can persist in soil for decades, contributing to long-term organic matter and improving nutrient and moisture retention [77]. Additionally, they enhance soil porosity and aggregate stability, promoting water infiltration, root penetration, and cation exchange capacity (CEC) [78]. By retaining moisture, biochar and hydrochar can alleviate drought stress, while improving water movement and nutrient availability helps mitigate soil salinity [79,80]. Hydrochar typically contains higher levels of water-soluble nutrients such as nitrogen, phosphorus, and potassium, providing more immediate benefits for plant growth [81]. Biochar application has been associated with variable but generally positive effects on crop productivity and soil quality. Meta-analyses indicate that biochar can increase crop yields by 5–51% and soil organic carbon by 12–102%, reflecting its potential for carbon sequestration. Biochar also reduces nitrate leaching and enhances ammonium retention, influencing soil nitrogen dynamics. Its effect on greenhouse gas emissions is inconsistent: methane (CH_4_) may be reduced by 9–72% and nitrous oxide (N_2_O) by 14–60%, although some studies report no significant change [82]. These variations are likely due to differences in management practices, soil type, and environmental conditions.

Winter cover crops are another sustainable strategy that improves soil health and mitigates abiotic stresses such as erosion, drought, and nutrient deficiencies [83,84]. Cultivating winter cover crops can enhance soil health and alleviate abiotic stresses by increasing organic matter content in the soil [85]. Organic matter plays a crucial role in improving soil structure, nutrient retention, and microbial activity. As cover crops develop, their roots penetrate the soil, contributing to organic material that decomposes and enriches it. Notably, certain winter cover crops, especially legumes, can fix atmospheric nitrogen, thereby enhancing soil fertility and reducing reliance on synthetic fertilizers [86]. Meta-analysis of 104 studies (1027 field-based yield records) indicated that cover crops generally increase subsequent main crop yields by 2.6%, although the effect varies widely depending on crop type, soil texture, tillage, and environmental conditions. Leguminous cover crops showed the greatest potential, raising yields by 9.8%, particularly when paired with corn, and up to 21.8% if the main crop was grown without additional fertilization. Cover crops on coarse-textured soils and in rainfed drylands increased yields by 14.1% and 11.4%, respectively. In fine-textured soils, conventional tillage with cover crops improved yields by 4.8%, whereas no-till systems with cover crops saw a 9.5% decrease [87]. Non-legume cover crops, short-term cover cropping, and introduction in no-till fine soils often impaired yields [88]. These data highlight that the benefits of cover crops are generally positive but highly dependent on nitrogen availability, soil moisture, and management practices. During winter, soils are frequently left bare or only lightly covered, rendering them susceptible to erosion from wind and water. The dense root systems of winter cover crops act as a protective barrier for the soil surface, mitigating erosion and preserving valuable nutrients. Additionally, the presence of cover crops enhances water infiltration and reduces surface runoff [89]. As these cover crops decompose in the spring, they release nutrients back into the soil in a form that is more readily available to plants, thereby improving nutrient cycling and minimizing the risk of nutrient imbalances. Furthermore, the root systems of winter cover crops improve soil structure by creating channels that facilitate deeper water penetration and access to nutrients from lower soil layers, which helps prevent salt accumulation in the topsoil [90,91]. The decomposition process of winter cover crops provides a consistent supply of organic matter, nourishing beneficial microorganisms such as bacteria, fungi, and earthworms, thereby enhancing soil biodiversity [92].

## 3. Multi-Omics Approaches for Enhancing Crop Resilience

### 3.1. Genomics and Genetic Engineering

#### 3.1.1. Genome-Wide Association Studies (GWAS)

**Figure 2:**
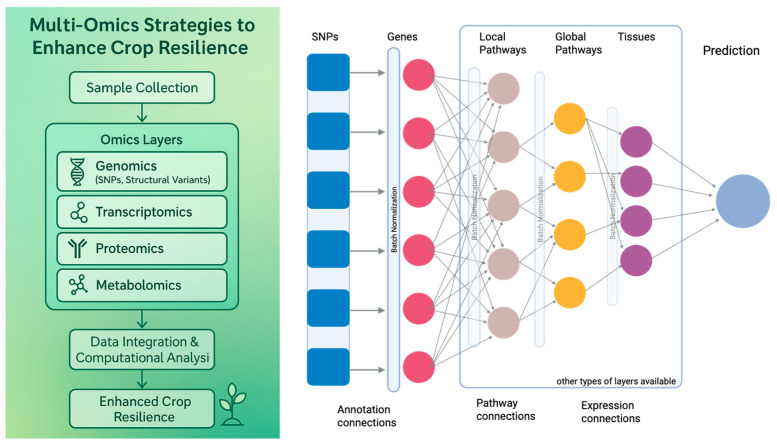
Integration approach of genomics, transcriptomics, proteomics, and metabolomics to enhance crop resilience to stress.

GWAS have emerged as a fundamental approach for identifying genetic loci associated with complex traits by examining genome-wide variation across diverse natural populations [93]. GWAS have developed into a potent tool for improving crop yield prediction. In intermediate wheatgrass, incorporating GWAS-identified 154 loci for key yield traits, and incorporating significant SNPs as fixed effects in genomic selection models improved predictive ability by up to 14% [94]. Similarly, in maize, using GWAS-derived SNPs improved prediction accuracy by 1.43–40% for kernel row number and 1.37–22.41% for ear length, depending on the model and trait complexity [95]. For crop stress tolerance studies, GWAS enables the systematic association of single-nucleotide polymorphisms (SNPs) with key agronomic traits such as drought endurance, salinity resistance, and heat tolerance [96,97,98]. In durum wheat, a panel of 208 lines genotyped with 6211 DArTseq (Diversity Arrays Technology sequence) SNPs showed mean yield reductions of 60% under drought (2.33 t/ha) and 72% under heat (1.64 t/ha) relative to yield potential (5.79 t/ha), with heat stress yields 30% lower than drought. GWAS revealed significant associations on chromosomes 2A and 2B (*p* = 10^−6^–10^−3^) explaining 7–25% of variation, with overlapping loci across stress indices (2B under drought; 3B and 7A under heat) and QTL hotspots on 2A (54–70 cM) and 2B (75–82 cM), demonstrating its effectiveness for mapping abiotic stress tolerance [94]. With engaging high-density genotyping platforms and extensive phenotypic datasets, GWAS provides insights into the polygenic nature of stress responses and uncovers allelic variants contributing to resilience. In maize, GWAS identified quantitative trait loci (QTLs) related to drought tolerance with the proportion of tolerant genes ranging from 53.3% to 86.7% across five drought-resistant inbred lines [99], emphasizing mechanisms of osmotic adjustment and root architecture modulation [100,101]. Similarly, in rice, 20 SNP loci associated with the sodium-potassium ratio have been identified, showing phenotypic variation ranging from 5% to 8% and a key QTL on chromosome 1 that is involved in salinity tolerance, notably sodium and potassium homeostasis [102,103]. Moreover, GWAS has been instrumental in identifying candidate genes encoding transcription factors such as DREB (Dehydration-Responsive Element Binding), NAC (NAM: no apical meristem; ATAF: Arabidopsis Transcription Activation Factor; and CUC: Cup-Shaped Cotyledon), and transporters including HKT1 that mediate stress adaptation [104,105]. These identified markers are invaluable for marker-assisted selection [MAS] and genomic selection (GS), facilitating early and efficient breeding of stress-tolerant varieties [106]. The integration of GWAS findings with functional genomics, such as transcriptomics (Figure 2) and CRISPR-based validation, further accelerates the translation of genetic discoveries into resilient crop cultivars.

#### 3.1.2. CRISPR and Gene Editing for Stress Tolerance

CRISPR-Cas9 technology has revolutionized plant genetic engineering by enabling precise, efficient, and targeted modifications of plant genomes, providing a transformative platform for improving stress tolerance traits [107]. Through site-specific genome editing, CRISPR-Cas9 enables the knockout, insertion, or replacement of genes associated with plant stress responses, thereby facilitating the development of crops that are better suited to withstand environmental challenges. CRISPR-targeted editing of key transcription factor genes, including DREB and NAC families, has been demonstrated to enhance resilience to abiotic stresses, including drought, salinity, and heat [108,109,110]. In drought conditions, *GmNAC12* overexpression in soybean improved survival rates by over 57% compared to wild type, whereas knockout lines showed at least a 46% reduction in survival [109]. Similarly, CRISPR/Cas9 knockout of *OsNAC041* in rice increased plant height but caused salt sensitivity, highlighting NAC genes as key targets for breeding stress-resilient crops [110]. Moreover, CRISPR/Cas9 knockout of *OsERF922* in rice significantly enhanced blast resistance [111], supporting earlier findings that this transcription factor negatively regulates both blast resistance and salt tolerance [112]. A recent study on targeted knockout of related susceptibility genes, including *OsERF922* homologs, has revealed alternative genetic sources of resistance to *Pyricularia oryzae* [113]. *OsRR22*-edited lines exhibited enhanced salinity tolerance, with shoot fresh weight reductions as low as 2.1%, compared to 50.3% in wild type [114]. Targeting *OsSAP* under drought led to 2.1–2.9 times higher 1000-grain weight [115]. Additionally, CRISPR-Cas9 gene editing of the grain size regulator GS3 and grain number controller Gn1a, three novel rice genotypes (*gs3-N9108*, *gs3-Z22*, and *gs3gn1a-Z22*) were developed that demonstrated consistent yield advantages of 3–7% over wild-type plants in field evaluations [116]. This technology has shown measurable improvements in rice performance under stress conditions. Similarly, in wheat, editing of ABA signaling components, including *TaPYL1*, has led to enhanced drought resilience through modulation of stomatal behavior and water-use efficiency [117]. Beyond gene knockouts, CRISPR-Cas9 facilitates base editing and prime editing, enabling the precise introgression of favorable alleles from wild relatives into elite cultivars without linkage drag, thus broadening genetic diversity and enhancing crop adaptability [118,119]. Furthermore, multiplex genome editing allows simultaneous modification of multiple genes, a strategy increasingly important for engineering complex traits such as combined drought and heat tolerance [120]. Overall, CRISPR-based genome editing holds enormous potential to accelerate the breeding of climate-resilient crops in an era of increasing environmental instability.

### 3.2. Transcriptomics: Gene Expression Under Stress

#### 3.2.1. RNA Sequencing (RNA-Seq) for Identifying Stress-Responsive Genes

RNA-Seq has emerged as a powerful tool to capture dynamic transcriptional changes in plants subjected to environmental stress, enabling the identification of key regulatory genes that are activated or suppressed in response to specific cues [121,122]. The technique provides higher sensitivity and broader coverage than microarrays, allowing the detection of novel transcripts and low-abundance RNAs that are critical for stress responses [121]. In rice, RNA-Seq analysis revealed that the number of differentially expressed genes (DEGs) between two cultivars, N-22 and IR64, was minimal in roots (~3%) but substantially higher in leaves (over 41%). Comparisons of direct-sowing versus transplanting conditions further identified DEGs enriched in photosynthesis, nitrogen metabolism, and hormone signaling pathways, highlighting both tissue-specific and environment-dependent transcriptional plasticity [122]. Correspondingly, stress-induced transcription factors have been shown to regulate secondary metabolism and stress defense; in Arabidopsis, members of the myeloblastosis (MYB), Basic Helix-loop Helix (bHLH), WRKY amino acid motif (WRKY), and NAC families were identified as central regulators linking metabolic reprogramming with abiotic stress tolerance, directly influencing the biosynthesis of protective metabolites such as flavonoids and alkaloids [123]. In soybeans, RNA-Seq analysis identified a total of 2724 and 3498 DEGs, with 289 TFs, including Ethylene Response Factors (ERFs), bHLH, MYB, NAC, and WRKY families, being differentially expressed under drought stress, and 271 TFs under flooding stress, highlighting the complexity of stress management mechanisms [124]. Similarly, in Arabidopsis under salt stress, transcriptomic analyses have shown the activation of LEA45, GSTF6, DIN2/BGLU30, TSPO, GSTF7, LEA18, HAI1, ABR, and LTI30 [125]. In wheat, RNA-Seq under heat stress conditions has identified heat shock proteins [HSPs] and transcription factors like HsfA2 as critical components of thermotolerance [126]. Furthermore, time-course RNA-Seq studies enable the dissection of early versus late stress responses, offering temporal resolution to gene regulatory networks [19]. Overall, RNA-Seq provides a foundational resource for functional genomics, facilitating the identification of candidate genes and regulatory pathways for molecular breeding and genetic engineering aimed at enhancing crop stress resilience.

#### 3.2.2. Regulatory Networks in Plant Stress Adaptation

Beyond the analysis of individual genes, transcriptomics enables the construction of gene regulatory networks (GRNs) that model the complex interactions among transcription factors (TFs), non-coding RNAs (ncRNAs), and their downstream target genes [127,128]. GRNs provide a holistic framework to understand how plants coordinate diverse molecular pathways during stress responses. Key TF families such as WRKY, MYB, NAC, DREB, and bZIP frequently emerge as central nodes, coordinating the transcriptional activation or repression of multiple stress-responsive genes [123,129,130]. WRKY TFs are known to modulate genes involved in oxidative stress responses and pathogen defense, while MYB proteins regulate osmotic balance and secondary metabolite biosynthesis during abiotic stress. Moreover, the role of small non-coding RNAs, particularly microRNAs (miRNAs), has been increasingly recognized. miRNAs such as miR398 and miR169 are pivotal regulators of stress adaptation, mediating the cleavage or translational repression of transcripts encoding key signaling components [131,132]. Long non-coding RNAs (lncRNAs) also contribute by acting as molecular sponges or scaffolds to modulate gene expression under stress conditions [132,133]. By integrating transcriptomic data with bioinformatic network analyses, researchers can decipher hierarchical regulatory relationships and feedback loops, offering a systems-level perspective of plant stress biology (Figure 2). Such comprehensive network models are invaluable for determining master regulators and hub genes, which represent promising targets for genetic engineering aimed at enhancing crop resilience.

### 3.3. Proteomics and Metabolomics

#### 3.3.1. Stress-Induced Changes in Protein Expression

Proteomics provides a powerful platform to analyze global changes in the protein landscape of plants under stress conditions, offering direct insights into functional responses that are not always predictable from transcriptomic data alone. Stress exposures, such as drought, salinity, and heat, often result in significant shifts in protein abundance, localization, and post-translational modifications [134]. Key commonly identified stress-responsive proteins include HSP70 and HSP90, antioxidant enzymes such as superoxide dismutase (SOD) and catalase (CAT), and molecular chaperones that assist in protein folding and stabilization under adverse conditions [135,136]. Proteomic investigations of drought-stressed soybean have revealed upregulation of ion transporters, ROS-scavenging proteins, LEA proteins, and enzymes involved in osmotic adjustment, such as proline synthetase [137,138]. Furthermore, proteomics has proven instrumental in uncovering stress-induced post-translational modifications, including phosphorylation, ubiquitination, and sumoylation, which critically regulate protein stability, activity, and interactions during stress responses [139,140]. For instance, phosphorylation of signaling kinases such as MAPKs plays a crucial role in transducing environmental signals into appropriate cellular responses. Spatial proteomics can aid in studying complex protein–protein interactions [141] and offer several advantages over traditional methods, such as Cytotrap [142]. Advanced quantitative proteomic techniques, such as iTRAQ and label-free LC-MS/MS, have further enhanced the ability to detect subtle but significant changes in stress-related proteins, thereby deepening our understanding of the dynamic proteome remodeling that underpins plant resilience mechanisms [143].

#### 3.3.2. Metabolic Pathways Involved in Plant Defense

Metabolomics serves as a powerful approach for characterizing the comprehensive biochemical shifts that underpin plant survival and adaptation under environmental stress. Modern plant metabolomics typically employs high-throughput analytical platforms such as gas chromatography-mass spectrometry (GC-MS), liquid chromatography-mass spectrometry (LC-MS), capillary electrophoresis-mass spectrometry (CE-MS), and nuclear magnetic resonance (NMR) spectroscopy to detect, quantify, and annotate a wide array of primary and secondary metabolites [144]. These untargeted and targeted approaches enable holistic or pathway-specific insights into metabolic reprogramming. Advanced computational tools and multivariate statistical techniques, often combined with machine learning (ML), are used to identify key metabolite patterns and biomarkers of stress [145]. Exposure to stresses such as drought, salinity, and heat often leads to the accumulation of compatible solutes, including proline, glycine betaine, and soluble sugars, which help maintain cellular osmotic balance and stabilize proteins and membranes [146,147]. Antioxidants such as glutathione, ascorbate, and tocopherols are also upregulated to mitigate the damaging effects of reactive oxygen species (ROS) generated under stress conditions [148,149]. In addition to primary metabolites, stress conditions stimulate the biosynthesis of a wide range of secondary metabolites, including flavonoids, terpenoids, and phenolic compounds, which contribute to ROS scavenging, pathogen defense, and modulation of plant signaling pathways [150,151]. Hormonal reprogramming is another hallmark of stress responses, with elevated levels of abscisic acid (ABA), jasmonic acid (JA), and salicylic acid (SA) fine-tuning growth regulation, stomatal behavior, and defense activation [152,153]. Recent integrated metabolomic and transcriptomic studies have illuminated key metabolic shifts, such as the enhanced flux through the phenylpropanoid pathway and tricarboxylic acid (TCA) cycle under stress, highlighting critical nodes for potential genetic or chemical intervention [154,155]. Novel spatial metabolomics and single-cell metabolomics approaches are also being utilized to resolve cell-specific metabolic heterogeneity, offering new opportunities to dissect localized stress responses [145,156]. Additionally, the integration of metabolomics with GWAS (mGWAS) and metabolic QTL (mQTL) mapping has begun to elucidate genetic determinants of metabolic traits, providing molecular markers for breeding stress-resilient crops [157,158]. Thus, metabolomics not only provides a snapshot of plant physiological status but also uncovers potential metabolic engineering targets for improving crop resilience.

### 3.4. Epigenomics and Environmental Adaptation

#### 3.4.1. Role of DNA Methylation in Stress Memory

Epigenomic modifications, particularly DNA methylation, represent crucial mechanisms that regulate gene expression without altering the underlying DNA sequence. These modifications play a pivotal role in plant responses to environmental stress, influencing gene expression patterns and contributing to plant adaptation [159,160]. DNA methylation, through the addition of a methyl group to the 5′ position of cytosine residues, can silence or activate genes involved in stress responses, often without permanent genetic changes [161]. Exposure to various stressors, including drought, salinity, and heat, has been shown to induce stable or transient changes in DNA methylation patterns at key regulatory loci [162,163,164]. These epigenetic alterations enable plants to “remember” previous stress events, which results in faster and more robust activation of defense mechanisms upon re-exposure to similar stress conditions, a phenomenon often referred to as “stress memory” [165,166,167]. For example, in wheat and rice, DNA methylation changes induced by drought stress were found to enhance recovery and survival following repeated drought cycles, indicating that these epigenetic modifications may contribute to improved resilience and long-term stress tolerance [168,169]. These findings highlight the potential of targeting epigenomic pathways to improve crop stress tolerance and ensure better yield stability in the face of increasing environmental variability.

#### 3.4.2. Transgenerational Adaptation Through Epigenetic Modifications

Emerging evidence demonstrates that environmentally induced epigenetic changes can be inherited across generations, enabling plants to adapt to stress without altering their DNA sequence [166]. Epigenetic modifications, including DNA methylation, histone modifications, and small RNA profiles, can be passed down to offspring, effectively “priming” them for enhanced resilience to environmental challenges [170]. Such modifications can enhance the plant’s ability to cope with stress, even in the absence of genetic mutations. In Arabidopsis, stress-induced transgenerational responses specifically depend on altered DNA methylation of Dicer-like (DCL) 2 and DCL3, and small RNA (smRNA) silencing pathways [166]. Transgenerational epigenetic inheritance has been reported in Arabidopsis and rice, where progeny of stressed parents shows enhanced tolerance to drought, salinity, and other stresses [168,171]. In rice, multi-generational drought stress generated thousands of DMRs, with 25.94% of hypo-methylated (462/1781) and 30.15% of hyper-methylated (515/1708) DMRs inherited in Huhan3, and 35.87% of hypo-methylated (514/1433) and 22.00% of hyper-methylated (1460/6636) DMRs transmitted in II-32B, correlating with drought-responsive gene expression changes [171]. Previous studies have also shown that exposure to salt and osmotic stress in Arabidopsis leads to DNA methylation changes that are inherited by the next generation, conferring enhanced tolerance without modifications to the primary sequence of the genome. Exposure to salt and osmotic stress can induce heritable epigenetic changes, including DNA methylation and small RNA-mediated regulation, which enhance stress tolerance in progeny. In Nicotiana benthamiana, infection with Potato virus X (PVX) or a chimeric PPV-P25 virus—but not PPV alone—conferred transgenerational tolerance, which was also observed in progeny of DCL3 mutants, highlighting the role of 24-nt siRNAs in mediating this inherited stress adaptation. [172]. Similarly, in rice, salinity-induced epigenetic changes are passed on to offspring, improving their resilience to salinity stress [173]. The stability and mechanisms underlying transgenerational epigenetic inheritance are still being elucidated; however, epigenetic engineering opens new avenues for crop breeding strategies aimed at enhancing stress tolerance [174]. Through the use of natural epigenetic processes, it may be possible to precondition crops to better withstand the increasing frequency and intensity of environmental stresses brought about by climate change.

## 4. Field-Based Phenomics: Advanced Trait Monitoring

### 4.1. High-Throughput Screening Technologies

Field-based phenomics, powered by advanced high-throughput phenotyping [HTP] technologies, is revolutionizing modern crop monitoring. The growing challenges of food security and climate resilience emphasize the need for innovative, data-driven solutions in agriculture. By integrating cutting-edge sensing technologies with ML, HTP enables large-scale, precise trait monitoring and facilitates genetic improvement and agronomic decision-making [175]. HTP relies on diverse sensor technologies, including red, green, and blue (RGB), multispectral, hyperspectral, Light Detection and Ranging (LiDAR), and thermal sensors [176]. Each component plays a vital role in capturing plant morphology, canopy architecture, chlorophyll content, and stress responses with high precision. LiDAR-based 3D imaging further enhances structural analysis, allowing detailed measurements of plant height and biomass accumulation with minimal environmental disturbance [177]. A ground-based LiDAR-equipped autonomous platform (DairyBioBot) demonstrated high accuracy in estimating perennial ryegrass biomass, achieving strong correlations with fresh mass (R^2^ = 0.71 at row level and R^2^ = 0.73 at plot level). These results highlight the effectiveness of LiDAR in providing non-destructive, high-throughput biomass assessments for forage breeding programs [178]. RGB, multispectral, and thermal imaging enhance the estimation of important crop parameters such as yield, abiotic stress, and canopy height [179]. A recent study shows that UAV-based RGB imaging has demonstrated strong accuracy in estimating plant height across growth stages, with R^2^ values up to 0.95 and RMSE ≤ 4 cm, highlighting its effectiveness and scalability in breeding trials [180]. A comparative study demonstrated that thermal cameras, particularly the ICI 8640 P, are highly effective for plant phenotyping, with the ICI showing the highest heritability value (0.756) for plot mean temperatures and image quality metrics compared to FLIR and thermoMap, indicating strong genetic signal capture in thermal imaging [181]. Additionally, hyperspectral imaging (HIS) provides high-resolution spectral data for plant phenotyping, nutrient content estimation, stress, and disease detection [182]. A UAV-based HSI system achieved an R^2^ of 0.79 for yield prediction at sub-plot level in wheat trials, demonstrating its effectiveness for high-throughput breeding decisions [183]. Combining HSI with deep learning models has shown the potential to identify crop diseases before visible symptoms appear, improving response times and reducing yield losses [184]. These sensor technologies onboard uncrewed aerial vehicles (UAVs) and uncrewed ground vehicles (UGVs) have expanded the capacity for rapid, cost-effective data collection over large experimental plots, offering new opportunities for non-invasive crop assessment in field conditions [185]. Despite these advancements, challenges persist in standardizing data collection protocols and ensuring cross-platform interoperability. The vast amount of data generated by HTP systems necessitates robust computational tools and cloud-based storage solutions to streamline analysis and accessibility. Additionally, transforming phenotypic data into actionable insights requires close collaboration between geneticists, agronomists, agricultural engineers, and data scientists to optimize breeding strategies and field management practices [185]. By leveraging advanced phenotyping technologies and AI-powered analytics, researchers can accelerate genetic improvements, enhance crop resilience, and promote sustainable agricultural practices for future food security [175].

### 4.2. Integrating Multi-Omics and Phenomics

ML, a subset of AI, focuses on developing algorithms that use statistical and computational techniques to “learn” from data and make predictions or decisions without being explicitly programmed [186]. This data-driven approach enables the analysis of complex patterns, making ML especially valuable for predictive modeling in biological systems [187]. Once a set of patterns is learned, the model can predict a broad spectrum of outputs. Generally, ML methods are categorized into supervised and unsupervised learning [188]. A promising subgroup of ML, deep learning (DL), is based on artificial neural networks with multiple layers, offering advanced capabilities that automatically extract features from raw data. Unlike traditional ML methods that rely on labor-intensive pre-processing engineering pipelines and visible feature extraction through data transformation, DL autonomously learns characteristics or patterns [189], making them highly effective for analyzing large-scale biological datasets. DL models have also been developed to predict complex traits from genomic data, facilitating faster and more accurate breeding decisions. SoyDNGP is a DL framework designed to predict phenotypes such as flower color and seed weight in soybean using SoyBase, a genotype database [190]. The model utilizes a convolutional neural network (CNN) architecture that captures both spatial and mutational context, achieving high prediction accuracy. The model is also adaptable to other crops like rice, maize, and tomato, highlighting its cross-species scalability. Similarly, RiceSNP-BST and RiceSNP-ABST are DL models designed to predict biotic and abiotic stress-related phenotypes in rice from GWAS data [191,192]. These models use automated CNN architecture searches and employ diverse feature encoding techniques such as One-hot, DNA2vec, DNABERT, and DNAshape to capture both sequence and structural information. They also enhance interpretability through tools like SHAP (SHapley Additive exPlanations), which assigns importance values to input features, and causal learning, which identifies key DNA structural attributes contributing to SNP function and transcription factor binding [193,194]. These capabilities make such models valuable tools for precision breeding under changing environmental conditions. While these technologies show significant promise, their implementation in sustainable agriculture must be assessed based on several criteria, including predictive accuracy, interpretability, scalability across species, data requirements, computational cost, and biological relevance.

Traditional GWAS identifies genetic associations but often lacks mechanistic explanations. While AI-driven models improve prediction by integrating multi-omics data, their limited interpretability hinders biological insights. To address this, interpretable AI is becoming increasingly important in GWAS and phenotype prediction, enhancing transparency and providing deeper insights into complex biological processes. GenNet [195] is a recently used open-source deep learning framework designed for interpretable phenotype prediction from genetic variants in human datasets, applied to seventeen phenotypes, including hair and eye color, as well as schizophrenia, with high accuracy. It offers more interpretability and easier understanding in genetic studies. Gennet Framework constructs biologically informed neural network architectures by defining sparse, hierarchical connections between layers as SNPs to genes, and genes to pathways or tissue types based on curated annotations from databases such as NCBI RefSeq, KEGG, and GTEx. Architecture allows for interpretation across layers, making GenNet a valuable tool for discovering functional insights into the genetics of complex traits [195]. While recently applied in human GWAS and phenotype prediction, it holds significant potential for future applications in plant GWAS and phenotype prediction. Its application in plant science could significantly improve breeding efficiency, trait selection, and precision agriculture, ultimately contributing to the development of more resilient and high-yielding crop varieties. The combination of multi-omics techniques has been transformational in terms of agricultural production prediction and genetic gain. In rice hybrid breeding, combining genomic, transcriptomic, metabolomic, and phenomics data significantly improved predictability compared to single-omics models, with yield-related traits seeing significant increases: grain yield predictability increased by 13.6%, tiller number by 54.5%, grains per panicle by 19.9%, and 1000-grain weight by 8.3% [196]. Similarly, in maize, machine learning models that incorporate genomic and environmental data (e.g., Random Forests, XGBoost) improved predictive accuracy for genotype-by-environment interactions by 7–11% compared to traditional methods, allowing for more precise selection of high-performing hybrids [197].

## 5. The Role of AI and ML

### 5.1. AI in Data Analysis and Prediction

AI transforms data analysis and prediction across multiple disciplines, offering unprecedented capabilities in extracting meaningful insights from complex datasets. In agriculture, AI-driven methodologies enhance predictive modeling, optimize resource allocation, and accelerate breeding advancements by efficiently analyzing vast amounts of genetic, environmental, and phenotypic data [198]. Advanced ML algorithms, including deep learning and ensemble methods, have demonstrated remarkable success in predicting complex biological outcomes. For instance, AI-driven models have significantly improved the accuracy of yield forecasting and stress tolerance assessments in crops, outperforming conventional statistical techniques [199]. The ability of AI to process large-scale genomic data further enhances the efficiency of trait selection, reducing the need for extensive field trials while maintaining high predictive reliability [200]. In predictive modeling, AI refines data processing by identifying key variables and their interactions, enabling the optimization of breeding strategies and environmental adaptation. Convolutional neural networks and support vector machines have been successfully applied in HTP, automating the classification of plant traits and stress responses from imaging data [201]. These advancements streamline decision-making by providing real-time insights into crop performance, allowing researchers to develop adaptive strategies for improving agricultural resilience [198]. Despite its transformative potential, AI-driven data analysis faces challenges related to model generalization and interpretability, data standardization, and computational demands. The complexity of integrating heterogeneous datasets from genomics, phenomics, and environmental monitoring necessitates robust analytical frameworks and interdisciplinary collaboration [199]. AI technologies continue to evolve, and their integration into predictive modeling will further refine analytical precision, fostering advancements in diverse fields from agriculture and beyond [198]. Furthermore, recent research has demonstrated that AI frameworks can select the most suitable ML algorithm for specific agricultural datasets, achieving prediction accuracies of 89.38%, 87.61%, and 84.27% for decision tree, random forest, and random tree models, respectively, thereby enhancing computational efficiency and scalability in smart farming systems [202]. Additionally, AI and IoT-based models that utilize real-time environmental sensor data have shown high accuracy in crop recommendation tasks, with Naïve Bayes classifiers achieving up to 99.39% prediction accuracy, highlighting the strong potential of supervised learning in smart agriculture applications [203].

### 5.2. AI in Precision Agriculture

**Figure 3:**
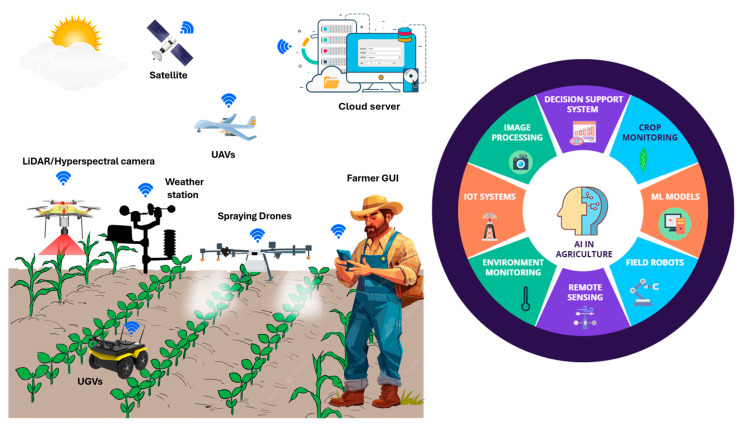
Visual illustrations of the role of artificial intelligence in agriculture: monitoring, analysis, and precision agriculture.

Food security and maximizing crop production are growing challenges in the agriculture sector. Precision agriculture was driven to address spatial and temporal field variability while achieving economic savings and environmental benefits, and it has been transformed by AI technologies. The integration of AI with precision agriculture has resulted in a transformative shift in modern farming with enhanced crop monitoring and management and increased automation to accommodate these challenges [204]. The integration of AI with decision-support systems in precision agriculture is revolutionizing farm management by leveraging real-time data collected through Internet of Things (IoT)-based sensor systems [205]. IoT enables the storage of essential sensor data and crop management information in a centralized cloud system, enhancing cost efficiency and computational power [206]. These systems have the potential to be expanded with the integration of different platforms like UAVs and UGVs. Integrating UAVs and UGVs with different sensing technologies marks a significant breakthrough in robotics. UAVs can monitor vast agricultural fields from the air, detecting crop stress caused by pests, diseases, or water deficiencies, and relay this data to UGVs, which then execute precise interventions such as fertilization, pesticide applications, or irrigation [207]. The combination of satellite imagery, UAVs, UGVs, ground-based sensors, IoT-based systems, and AI algorithms equips farmers with real-time data, allows proactive decision-making in precision agriculture [208]. Recent European projects have demonstrated the effectiveness of AI in optimizing precision agriculture through advanced data fusion, real-time decision-making, and predictive analytics. For example, the AI-enhanced QUHOMA irrigation system reduced water use by 6–11% in strawberry crops, while the VINBOT and DEMETER projects applied ML to predict vineyard and olive yields based on canopy features and phenological data. These implementations confirm AI’s role in improving input efficiency, yield forecasting, and sustainability in modern agriculture [209]. A summary of the modern agricultural system is illustrated in Figure 3.

### 5.3. AI and Automated Crop Management

The use of AI in crop management is reshaping modern agriculture, driving improvements in efficiency, sustainability, and resilience [210]. These digital technologies can boost productivity by optimizing the management of inputs such as irrigation, seed, nutrients, and pesticides to mitigate the issues of pests and diseases. In agriculture, plant protection plays a crucial role in maintaining crop health and boosting yields. Intelligent pesticide prescription spraying systems observe and identify pests, diseases, and weeds, utilize data-informed strategies to develop tailored precision management plans, and employ smart tools to take preventive actions [211]. Recent developments in remote sensing and image analysis technologies have made it possible to accurately, instantly, and non-invasively identify pests and pathogens over extensive regions [212]. The use of AI-driven management strategies coupled with diverse sensing technologies can support the execution of disease control plans by leveraging early data on crop health and the precise location of diseases [213]. Common AI-based approaches for disease control include decision trees, random forests, k-nearest neighbors, support vector machines, and artificial neural networks [214]. Efficient irrigation and nutrient management are essential components of automated crop management. AI offers cutting-edge solutions to maximize resource use, improve crop health, and decrease environmental damage. Recent studies demonstrate that AI-integrated precision agriculture technologies, such as smart irrigation and sensor-based nutrient management, can reduce water use by 30–50% and increase crop yields by 10–30% [215]. Through the application of AI methods, farmers can improve irrigation and nutrient management, leading to increased yields, minimized resource use, and a smaller ecological impact [216]. AI algorithms can aid farmers in making better-informed choices, thereby minimizing the risks tied to climate variability and market changes.

The implementation of AI and ML technologies in agriculture requires a comprehensive evaluation framework to ensure their effectiveness and reliability. This framework should incorporate key performance dimensions such as predictive accuracy, generalizability, and scalability. Predictive accuracy is assessed using statistical metrics such as the coefficient of determination (R^2^), F1 scores and root mean square error (RMSE), by comparing model outputs with ground-truth observations [217]. Generalizability refers to the model’s robustness across varying agroecological conditions and can be evaluated by applying AI models to different crops, regions, and seasonal datasets. Scalability is demonstrated through the successful deployment of these models in large-scale farming operations, often facilitated by integration with platforms such as UGVs and UAVs.

## 6. Plant-Associated Microbiomes and Sustainable Agriculture

### 6.1. Microbiome Engineering for Stress Tolerance

#### 6.1.1. Role of Beneficial Microbes in Drought and Salinity Resistance

Beneficial microbes, particularly plant growth-promoting rhizobacteria (PGPR), are key to microbiome engineering for enhancing plant tolerance to drought and salinity stress, which threaten over 40% of global agricultural land and are increasingly intensified by climate change [218,219,220,221]. It may not be the case that a specific PGPR has a unique beneficial activity for either drought or salinity stress. Instead, it is likely that a beneficial activity of PGPR contributes to tolerance for both stresses, although certain activities play a more crucial role for specific stresses. It has been reported that diverse PGPR can promote the resilience of crops to drought and salinity through multifaceted mechanisms. Evaluation of PGPR efficacy under drought and salinity stress typically encompasses a few broad categories like plant physiological performance, biological stress indicators, molecular level adaptations, and integrated soil health improvements. In maize, Bacillus megaterium enhances root hydraulic conductivity under salinity, while Pantoea agglomerans increases root conductance [222,223]. Certain Bacillus and Pseudomonas species improve drought and salinity tolerance by producing 1-aminocyclopropane-1-carboxylic acid (ACC) deaminase, solubilizing phosphate, and secreting siderophores [224,225,226,227,228]. Some PGPR secrete exopolysaccharides [EPS], forming biofilms that stabilize soil structure, improve moisture retention, and enhance microbial colonization [229]. Regulation of ion homeostasis and redox balance through the production of various antioxidizing enzymes and non-enzymatic antioxidants is also a crucial mechanism by which PGPR mitigate drought and salinity stresses [230,231,232,233,234,235,236]. These above mechanisms highlight PGPR as a sustainable strategy for improving plant adaptation to drought and salinity stress.

#### 6.1.2. Synthetic Microbial Communities for Crop Resilience

The identification of microbial strains capable of producing different products that can assist in plant growth under stress conditions can help in promoting sustainable agriculture, given the escalating climate change impacts [237]. Synthetic microbial communities (SynComs), a strategically designed multi-strain approach, represent a significant advancement in enhancing crop resilience against environmental stressors. Unlike single-strain inoculants, SynComs integrate functionally complementary microbes that interact synergistically to enhance nutrient acquisition, pathogen suppression, and abiotic stress tolerance through inter-microbial communication, such as quorum sensing and metabolite exchange [238,239]. The composition of a SynCom can be designed through experimental studies on microbial interactions or through computational approaches, such as genome-scale metabolic modeling (GEM), utilizing genomic databases of beneficial microbes to predict their metabolic roles in enhancing plant resilience [240,241]. SynComs strengthen plant resilience by utilizing metabolically diverse microbes. For instance, co-inoculation of *Paenibacillus mucilaginosus* and metal-resistant *Sinorhizobium meliloti* improved Cu uptake, enhanced soil microbial biomass, and increased antioxidant activity while reducing oxidative stress [240,242,243]. Additionally, co-culturing Pseudomonas koreensis and Microbacterium hydrothermale enhanced ACC deaminase, indole-3-acetic acid (IAA), and cytokinin production, reducing ethylene emissions by 20% and mitigating oxidative stress under salinity stress [244]; and a consortium of *Ensifer adhaerens*, Pseudomonas fluorescens, and Bacillus megaterium improved wheat resilience to salinity by enhancing shoot and root growth, chlorophyll content, and K/Na balance while reducing electrolyte leakage [245]. Combining multiple beneficial microbes can also lead to disease suppression and enhanced nodulation and nitrogen fixation. This is exemplified by mixtures of Bacillus spp. with species of Pseudomonas, Rhizobium, or Bradyrhizobium [246,247,248,249,250]. The integration of various microbial functions, such as ACC deaminase activity, osmolyte production, and biofilm formation, further enables SynComs to regulate phytohormones and improve stress adaptation. This approach supports SynComs’ role in promoting sustainable agriculture and developing climate-resilient crops [245,251]. Nevertheless, the successful application of SynCom largely depends on the survivability of microbes during preservation and in non-native soils. Development of innovative encapsulation and coating techniques is crucial for the widespread use of SynComs [252,253].

### 6.2. Bioinoculants and Soil Health Improvement

#### 6.2.1. Enhancing Nutrient Uptake and Root Development

Bioinoculants, primarily beneficial bacteria, play a crucial role in improving plant nutrition by facilitating nutrient uptake and optimizing root-system architecture (RSA), with their efficacy assessed through enhancements in soil nutrient availability, measurable root trait improvements, and overall economic viability. These microbial agents aid in nutrient solubilization, mobilization, and assimilation, thereby reducing dependency on synthetic fertilizers. Nitrogen-fixing bacteria, such as Rhizobium, Bradyrhizobium, Azospirillum, and Azotobacter, convert atmospheric nitrogen into ammonia, increasing nitrogen availability [254,255,256]. Phosphate-solubilizing bacteria, including Pseudomonas, Enterobacter, and Leclercia, produce organic acids that release phosphorus from insoluble compounds, improving its accessibility to plants [257,258,259]. Similarly, other nutrient elements can be better obtained through the action of beneficial bacteria, such as potassium by potassium-solubilizing bacteria [260,261], silicon by silicate-solubilizing bacteria like *Burkholderia eburnea* and *Bacillus globisporus* [262,263], iron by siderophore-producing PGPR, including Pseudomonas, Stenotrophomonas, and Enterobacter [264,265,266], manganese by certain Bacillus and Pseudomonas strains [267,268], and selenium (Se) by selenium-accumulating PGPR, such as Enterobacter and Bacillus [269,270]. The enhanced nutrient uptake mediated by bioinoculants helps mitigate various biotic and abiotic stresses. Bioinoculants also influence root morphology by producing phytohormones, such as IAA and gibberellic acid (GA). For example, application of bacterial culture supernatant of Ignatzschineria sp. has been shown to promote lateral root formation by up to 46%, root elongation by up to 18%, and root hair proliferation by up to two-fold compared to the untreated control [271,272]. This structural modification improves water and nutrient uptake efficiency, particularly under drought and salinity stress [273,274,275]. These microbes interact dynamically with plant roots by sensing and responding to root exudates, which regulate bacterial gene expression, metabolism, and colonization [276]. These interactions support microbial adaptation and plant resilience [277,278,279,280] and promote soil stability, organic matter decomposition, and microbial diversity, ultimately leading to sustainable agricultural practices and long-term soil health.

#### 6.2.2. Imaging Methods for Evaluation of Root System Architecture

RSA is composed of the overall spatial and morphological features of the root system of a plant, with these features related to phenotypic traits. Investigating how RSA changes in response to different factors allows greater understanding of the connections between plants and environment [281]. Measurements of RSA features is pursued through application of a variety of imaging techniques [282,283], commonly defined by specific methods of plant and root cultivation and potential root harvesting [284]. These methods may be viewed as choices along a spectrum from destructive to non-destructive [285]. Destructive methods involve those seen generally in field-size and some greenhouse experiments, including harvesting of the entire root system through use of “shovelomics”, where the root crown is removed [286,287,288], exposure of the root system of plants by trenching alongside [289], and traditional coring, with removal of cylindrical cores containing whole roots, pieces or subnetworks of roots [290]. At the greenhouse level, roots may be extracted from pots. Generally, roots and root materials are washed after harvesting, and this washing step itself may be destructive. Roots are then imaged with various systems, including 2D imaging with RGB or monochrome flatbed scanners, DSLR cameras, machine vision cameras, or minirhizotron tube cameras [after coring in the field] [291]. Three dimensional imaging techniques may be applied as well including multi-camera setups with sample revolution [292], with 3D reconstructions of the root system possible after image processing.

Nondestructive methods are seen at the greenhouse and bench levels, including use of various rhizotrons or rhizoboxes where roots are grown in boxes with visible windows [293,294], clear pots or boxes filled with soil [295], on germination paper [296], in agar filled Petri dishes or containers [295], or hydroponically [297], at varying levels of plant maturity, and length scale. Imaging in 2D is possible through transparent windows and sides with systems including RGB/monochrome DSLR and machine vision cameras. With these types of methods 3D imaging is also more accessible, including magnetic resonance imaging (MRI) [298], x-ray computed tomography [298,299,300], and positron emission tomography [283]. Laser light has also been used in conjunction with gel medium [301]. MRI and tomographic methods are affected by challenges in imaging resolution, soil material opacity, and water interactions, amongst others. Choice of imaging method for evaluating RSA is often dependent on experiment size and hardware, and on subsequent image processing pipeline decisions.

#### 6.2.3. Reducing Dependency on Chemical Fertilizers

The increasing reliance on synthetic fertilizers, particularly nitrogen-based formulations, has significantly contributed to soil degradation and greenhouse-gas emissions, accounting for approximately 2.1% of anthropogenic emissions [302]. Bioinoculants offer an environmentally friendly approach to enhancing plant growth and nutrient acquisition through improved microbial metabolic networks [303,304,305,306,307,308,309]. Evaluation of bioinoculant strategies includes gains in crop yield and nutrient use efficiency, reductions in synthetic fertilizer inputs and greenhouse-gas emissions, and economic viability at farm scale, factors directly relevant to climate-smart agriculture. The application of biofertilizers (or bioinoculants) has been shown to enhance crop yields by 25% while significantly lowering the need for synthetic inputs, reducing nitrogen requirements by 50% and phosphorus needs by 25%. This reduction not only minimizes dependency on chemical fertilizers but also decreases soil and plant vulnerability to pests and diseases, thereby lowering the need for synthetic pesticides [310]. The shift toward biofertilizers is reflected in market trends, with the U.S. biofertilizer market projected to grow from USD 3.55 billion in 2024 to USD 4.47 billion by 2026 [309]. This growth is driven by various advantages of using microbial agents, including the enhancement of plant resilience against diverse environmental stresses [311,312,313].

## 7. Societal, Social, and Economic Impacts of Climate-Resilient Crops

### 7.1. Global Food Security and Hunger Reduction

Since the beginning of the 21st Century, the world has seen substantial reductions hunger and food insecurity, but both hunger and food insecurity continue to be major social challenges in all world regions, and particularly in sub-Saharan Africa [314]. Future population, agricultural land use, and climate projections suggest that between 2.5 and 4.2 billion people (51% of the world’s population) will be at risk of undernourishment by 2050 without agricultural innovation for climate adaptation in the intervening years [315]. In the world’s most vulnerable regions, hunger, food insecurity, and rapid food price increases have been linked to social unrest and food riots, most recently in 2008 and 2010 [316]. Weather volatility and higher temperatures have reduced crop yields and contributed to a rise in food shortages, hunger and food insecurity, and undernourishment is predicted to increase in countries with increasing populations [317]. Climate-induced crop failure due to temperature increases and weather volatility has been a major contributor to both external and within-country migration, particularly in countries that rely more heavily on the agricultural sector [318]. Projected yield decreases from weather variability are greatest for oilseed crops, which include soybeans [319]. While intensive efforts in several other areas may also be needed to minimize the impact of extreme and volatile weather, learning more about the resilience of plants, soils, and microbial communities to improve crop yields in adverse and variable environmental conditions will constitute one crucial component of the global effort to meet reduce food insecurity [320]. The iPACERS project focuses efforts to understand biotic and abiotic stress tolerance of one of the most important food crops, soybeans (the King of Beans), one of the most important sources of protein for the world’s population with a global market value projected to increase from USD 155 billion in 2023 to USD 278 billion by 2031 [321].

In the United States, soybean production has increased over the last 100 years from about 1.5 million acres in 1924 to nearly 90 million acres in 2024 [322]. Soybeans are currently the second largest row crop in the United States, and if demand continues to grow, soybeans could replace corn as the most planted crop [323]. Extreme weather events can cause crop failure or significantly lower yields that can influence farming strategies and decision-making strategies, farmer mental health, suicide rates, and community participation. A deeper understanding of how extreme and volatile weather conditions affect soybean plants, root systems, and soil microbiomes at the cellular level can empower soybean farmers to develop and implement more effective adaptation strategies. Farmers who perceive that climate change has increased risks to their own farm are more likely to explore adaption strategies, experiment with a wider variety of adaption strategies, and take more advantage of available support structures, such as crop insurance programs, community support through grower associations, and conservation programs [324].

Changing weather conditions over the past decades has resulted in decreasing production of corn and cotton in Louisiana, along with increasing production in rice and soybeans, and these trends are likely to continue in the future, increasing the need for research that specifically focuses on adaptation strategies related to soybean farming [325]. Effective strategies require both deeper understanding of biotic and abiotic stress of the soybean plant under extreme or volatile weather conditions and the willingness of farmers to implement the strategies. A recent study partially attributed Michigan farmers’ willingness to adopt sustainable agricultural practices to both their understanding of the necessity and benefits of adopting a new variety or practice and their perceptions of the opinions and practices of other farmers in their social networks [326]. Concerns about climate change, crop failure, and lower yields can have negative consequences for farmers’ health and mental health [327] and suicide [328]. Advances in agricultural technologies and plant breeding have led to more climate-resilient crop varieties and more sustainable agricultural systems, even for smallholder farmers in developing nations, and show promise for additional mitigation strategies [329,330,331]. Climate-resilient crops can potentially reduce risks (real or perceived) to farmers due to weather volatility and extreme weather. Extension and support communities play a vital role in building relationships and engaging farmer stakeholders in the coproduction of scientific and technical knowledge to increase resilience in rural communities [332].

The iPACERS project attempts to address the complex challenges related to soybean production in the southern United States by integrating scientific knowledge related to soybean stress tolerance, root system dynamics, and soil microbiome responses to extreme weather with environmental educational outreach to farmers, teachers, and students in communities where soybean production has been established and is forecasted to expand in the coming decades. Participating scientists will help develop future generations of researchers through their respective labs and research programs. Importantly, the practical challenges faced by farmers and rural communities cannot typically be addressed using the theoretical perspectives and methods of a single academic discipline or institution. Therefore, multi-institutional and multidisciplinary approaches to the complex agroecological and social challenges related to climate change will continue to lead the efforts to find adaptation strategies that strengthen agricultural communities, mitigate the negative effects of extreme weather and weather volatility, build agricultural research capacity, and lead toward more sustainable agricultural systems. Scientists and their research programs benefit from these large-scale collaborations by gaining access to equipment, expertise, data, and techniques to conduct research and generate meaningful data and findings that have practical relevance for stakeholders. Stakeholder communities both benefit from and contribute to the broad dissemination of research findings, and these communities provide critical feedback that researchers can use to make advances in climate adaptation more accessible and relevant to local populations.

### 7.2. Integrating Climate-Agriculture Education

Climate change education enables individuals and communities to understand the impacts of climate change on agricultural systems and food security. It equips farmers, educators, policymakers, and students with knowledge and skills to mitigate climate change and adapt agricultural practices. Education has the potential to serve a critical role in addressing climate change by developing a population with climate literacy and offering potential solutions to mitigate the impacts of climate change [333,334,335]. A systematic review of environmental and climate education studies suggests that effective environmental education includes: (1) focusing on personally relevant and meaningful information and (2) using active and engaging teaching methods [336]. Specific to climate change, Monroe et al. (2019) also found four themes related to successful education initiatives: (1) engaging in deliberative discussions, (2) interacting with scientists, (3) addressing misconceptions, and (4) implementing school or community projects [336]. Projects that focus on local and individual action may be especially effective [335,337]. The authors of another systematic review of climate change education call for the development of a curriculum that “directly involves children and young people in responding to the scientific, social, ethical, and political complexities of the issue” [338]. An integrated, systems-based model for understanding both the impacts of climate change and the potential to develop solutions can make the topic both more meaningful and empowering for students [339,340].

Both the Next Generation Science Standards (NGSS), used in the majority of the United States to guide science education, and The National Council for Agricultural Education’s Agriculture, Food, and Natural Resources (AFNR) standards for K-12 agriscience education include standards related to understanding human impact on climate change and using biotechnology to adapt organisms (Table 1 and Table A1) [341,342]. However, neither set of standards has strong deliberate integration of the two ideas (i.e., using biotechnology to develop agricultural solutions to climate-change-driven problems), and in other parts of the world, there is a similar siloing of topics [343]. Teacher professional development (TPD) workshops could address this by training teachers in the use of CRISPR Cas-9 to develop drought-tolerant, heat-resistant, or other climate-change-resistant food crop plants. Commercial providers of K-12 biotechnology classroom kits have already begun to produce age-appropriate kits for classroom use. The AFNR standards could be an excellent resource for science educators to design biotechnology-driven climate education from, as they already consider the intersection of science, society, economics, and career applications. Our proposal of teaching climate change through the lens of agriscience and biotechnology solutions meets the extant literature’s suggestions for meaningful and effective classroom-based climate education.

School-based agriscience educators are an often-overlooked group in consideration of climate change education [344]. Local extension agents are also valuable as trusted sources of education and information for farmers [345]. Very few published studies have examined these educators’ knowledge and attitudes regarding climate change and those that have reported a range of knowledge, beliefs in the anthropogenic origins of climate change, and instructional time spent on the topic [346]. These educators should be considered for future research and professional development training. Among science teachers there is also a range of knowledge and beliefs regarding the inclusion of climate education in their curricula. For some teachers, teaching climate change aligns with personal beliefs about the importance of environmental education, their identity as scientists, and/or their feelings of hope in regard to solutions to climate-change-driven problems [347,348]. However, even among secondary science teachers who teach climate change, there may be misconceptions in their knowledge and a hesitancy to include social, political, economic contexts [349,350]. Future professional development opportunities should consider the range of potential educators, including science, agriscience, and Extension or other outreach teachers, the range of attitudes and beliefs, and the role that teacher science identity and actionable learning can play in effective climate education [351,352]. Integrating climate issues into biotechnology and agriscience education promotes sustainability and prepares future generations to tackle global challenges effectively. It also anchors a challenging and sometimes emotional topic in consideration of real-world impacts and engages learners in considering their ability to develop or support real, technology-driven solutions to global problems.

## 8. Future Directions

### 8.1. Integrating AI, Multi-Omics, and Phenomics in Crop Breeding

The integration of AI, multi-omics, and phenomics can reshape the landscape of modern crop breeding. According to McKinsey & Company, AI and advanced analytics in agriculture could contribute USD 500 billion annually to global GDP by 2030 through yield optimization and reduced losses [353]. Multi-omics approaches encompassing genomics, transcriptomics, proteomics, metabolomics, and epigenomics enable the comprehensive dissection of plant responses to environmental stresses at various biological levels. However, managing and interpreting the vast datasets generated from these methods requires sophisticated computational tools. AI and ML algorithms are being increasingly deployed to uncover complex genotype-to-phenotype relationships, predict trait performance, and guide marker-assisted and genomic selection processes [187,354]. ML models have been used to predict yield under drought conditions by integrating SNP markers with high-throughput phenotypic data [355]. Deep learning, a subset of AI, can also identify novel gene–trait associations from large omics datasets, enabling trait prediction with higher accuracy than conventional statistical methods. Simultaneously, advances in phenomics, especially HTP platforms using drones, imaging systems, LiDAR, and thermal cameras, allow precise, real-time monitoring of plant traits like canopy temperature, chlorophyll fluorescence, and water-use efficiency under field conditions [18,356]. Integrating these phenomic datasets with multi-omics profiles and AI-driven modeling enables predictive breeding, accelerates the identification of superior genotypes, and improves selection accuracy across diverse environments. For instance, the AI-driven CropQuant-Air phenotyping platform achieved exceptional performance in wheat yield classification using low-cost drone imagery. The system’s advanced canopy image analysis demonstrated classification accuracies of 97% for high-yielding varieties, 96.4% for medium-yielding, and 94.7% for low-yielding cultivars, enabling precise yield prediction through automated trait extraction [357]. The combination of these technologies paves the way of ‘data-driven breeding,’ where identifying climate-resilient varieties becomes more efficient, cost-effective, cost-effective, and more reliable.

### 8.2. Investment in Climate-Smart Agricultural Technologies

To translate scientific innovations into on-the-ground impact, substantial and sustained investment in climate-smart agriculture (CSA) is essential. CSA technologies aim to increase productivity, adapt and build resilience to climate change, and reduce greenhouse gas emissions [358]. CSA offers significant agronomic, economic, and environmental advantages. Farmers adopting CSA achieved a USD 259 increase in gross margins, representing a 69% return on investment, demonstrating its strong economic viability. Beyond profitability, CSA enhanced climate resilience by 18.5%, equipping farmers to better manage climate variability. Technologies such as zero tillage with residue retention, recommended fertilizer use, and advanced tools like Green Seeker, tensiometers, ICT-based advisories, and crop insurance significantly boosted productivity and profitability. Environmentally, CSA practices led to a 43% reduction in global warming potential, driven by lower CO_2_ and N_2_O emissions, and a 56% cut in energy-related GHG emissions, owing to improved energy efficiency and reduced fossil fuel use [359,360,361,362]. According to Challinor et al. (2014), agricultural adaptation strategies like CSA boosted crop yields by 7–15% compared to non-adaptive approaches [363]. In the Indo-Gangetic Plains, CSA implementation in rice-wheat systems significantly enhanced resource efficiency, increasing water productivity by 53.9% and energy productivity by 48.9%, while reducing irrigation water use by 39.3% [362]. These CSA technologies include precision agriculture tools, resource-efficient irrigation systems, stress-resilient crop varieties, decision support tools, and gene-editing platforms like CRISPR-Cas9. Public and private investment is particularly critical in accelerating the development and deployment of technologies that benefit smallholder farmers, who are disproportionately affected by climate variability [364]. Precision technologies such as smart irrigation controllers, moisture sensors, and AI-based pest forecasting systems are increasingly adopted in industrial agriculture but remain underutilized in resource-limited settings. CRISPR genome editing transforms crop improvement by enabling precise, rapid trait integration under stress with minimal off-target effects, warranting broader agricultural adoption [365]. Additionally, mobile-based advisory services and digital platforms are enhancing farmers’ access to climate and market information, empowering them to make informed decisions. Governments and development agencies must prioritize investments not only in technology but also in infrastructure, policy reform, and education. This includes supporting seed system development, strengthening biosafety regulations, and incentivizing private sector innovation. Importantly, investment must be inclusive, ensuring that women, indigenous communities, and marginalized groups benefit equitably from CSA advancements [366].

### 8.3. Strengthening Global Collaborations and Research Initiatives

The global nature of climate change and food insecurity calls for international collaboration and cross-sectoral partnerships. Strengthening global research networks can facilitate the sharing of germplasm, data, and best practices and reduce duplication of efforts. Major initiatives such as the CGIAR’s OneCGIAR reform, the FAO Global Soil Partnership, and the Crop Wild Relatives (CWR) Project represent coordinated efforts to mobilize international expertise and resources toward sustainable crop improvement [367]. According to a World Development study, CGIAR-developed crop technologies had achieved remarkable global adoption by 2020, covering 221 million hectares of farmland. These innovations generated substantial economic benefits, contributing approximately USD 47 billion in annual welfare gains and an impressive cumulative total of USD 1.334 trillion since CGIAR’s establishment in 1961 [368]. Collaborative breeding programs that integrate local knowledge with global science have shown success. For example, participatory breeding initiatives in Sub-Saharan Africa and South Asia have resulted in locally adapted, stress-tolerant varieties of rice, millet, and cassava. Similarly, North–South and South–South research alliances can enhance capacity building, particularly in developing nations facing the brunt of climate stress. To sustain and expand these efforts, policies must promote open data sharing, intellectual property transparency, and equitable access to genetic resources. Crop modeling has experienced renewed global attention, fueled by large-scale, multidisciplinary collaborations focused on advancing model frameworks. Leading programs like the Agricultural Model Intercomparison and Improvement Project (AgMIP), Global Futures & Harvest Choice, and the Climate Change, Agriculture and Food Security (CCAFS) initiative have driven key innovations. Recent breakthroughs include substantial upgrades to the ORYZA model, enhancing its precision in simulating rice growth under water- and nitrogen-limited conditions. In wheat modeling, a study evaluating 29 different models revealed that temperature response functions accounted for >50% of variability in grain yield predictions. Crucially, refining these functions in just four models reduced yield prediction errors by over 40% across seven globally distributed sites with diverse temperature regimes [369,370,371]. Training the next generation of plant scientists in interdisciplinary, collaborative, and globally minded frameworks will also be critical. Furthermore, establishing regional centers of excellence in climate-resilient agriculture can drive locally tailored solutions while feeding into global innovation pipelines. These centers should focus not only on scientific excellence but also on socio-economic impacts and policy translation.

## 9. Conclusions

The challenges to global agriculture posed by climate change demand urgent and innovative solutions. Integrating multi-omics, AI-driven analytics, and advanced phenotyping in developing climate-resilient crops can bring unprecedented developments. By deciphering complex stress responses and accelerating precision breeding, these technologies bridge the gap between research and real-world agricultural resilience. Harnessing plant–microbe interactions to enhance crop adaptability and soil health becomes possible. Beyond scientific advancements, the widespread adoption of these tools can drive socioeconomic progress through strengthening food security, empowering farmers, and fostering sustainable economies. Achieving this vision demands coordinated action among scientists, policymakers, and educators. The path ahead demands scalable, equitable implementation while advancing fundamental science to outpace evolving climatic challenges. Together, these strategies can safeguard food systems, ensuring productivity and sustainability in an uncertain climate future.

## Figures and Tables

**Table 1:** NGSS and AFNR Standards Related to Climate Change and Biotechnology.

Standards	Topic	
	Climate Change	Biotechnology
NGSS [341]	- Understanding human impact on the environment, including rising global temperature (MS-ESS: 3-3, 3-4, 3-5; HS-ESS: 3-4, 3-5, 3-6)	- Understanding genetic modification (MS-LS: 4-5) - Using genetic modification to minimize adverse human impact on biodiversity (HS-LS: 2-7, 4-6).
AFNR [342]	- Synthesizing environmental, sociopolitical, and cultural factors in resource management (NRS: 01.03, 02.01, 02.03) - Understanding and adjusting for effects of climate change on environmental sustainability systems (ESS: 03.01, 03.01.03.c, 03.01.04.c.) - Applying agricultural technology and policies at local, state, national, and global level (FPS.02.02, FPS.04.02)	- Plant propagation and impact of GMO crops (PS: 03.01, 03.01.05.c) - Using biotechnology to enhance plant production (BS: 03.04)

## Appendix A

**Table A1:** Supplementation of Table 1.

Standards	Topic	
	Climate Change	Biotechnology
NGSS [341]	- MS-ESS3-3. Apply scientific principles to design a method for monitoring and minimizing a human impact on the environment. - MS-ESS3-4. Construct an argument supported by evidence for how increases in human population and per capita consumption of natural resources impact Earth’s systems. - MS-ESS3-5. Ask questions to clarify evidence of the factors that have caused the rise in global temperatures over the past century. - HS-ESS3-4. Evaluate or refine a technological solution that reduces impacts of human activities on natural systems. - HS-ESS3-5. Analyze geoscience data and the results from global climate models to make an evidence-based forecast of the current rate of global or regional climate change and associated future impacts to Earth’s systems. - HS-ESS3-6. Use a computational representation to illustrate the relationships among Earth systems and how those relationships are being modified due to human activity.	- MS-LS4-5. Gather and synthesize information about technologies that have changed the way humans influence the inheritance of desired traits in organisms. - HS-LS2-7. Design, evaluate, and refine a solution for reducing the impacts of human activities on the environment and biodiversity. - HS-LS4-6. Create or revise a simulation to test a solution to mitigate adverse impacts of human activity on biodiversity.
AFNR [342]	- NRS.01.03. Apply ecological concepts and principles (e.g., weather, air quality, UV protection, atmospheric pressure, etc.) to the interaction of atmospheric and natural resource systems. - NRS.02.01. Examine and interpret the purpose, enforcement, impact, and effectiveness of laws, agencies, and private and public organizations related to natural resource management, protection, enhancement, and improvement (e.g., water regulations, game laws, environmental policy, local, state, and national conservation organizations, agricultural extension service, etc.). - NRS.02.03. Analyze how social perceptions of natural resource management, protection, enhancement, and improvement change and develop over time. - ESS.03.01. Apply meteorology principles to environmental sustainability systems. - ESS.03.01.03.c. Evaluate the potential impacts of global climate change on environmental sustainability systems. - ESS.03.01.04.c. Create an action plan to mitigate the impact of climate change on environmental sustainability systems. - FPS.02.02. Examine the impact of AFNR on the local, state, national, and global society and economy. - FPS.04.02. Assess and explain the natural-resource-related trends, technologies and policies that impact AFNR systems.	- PS.03.01. Demonstrate plant propagation techniques in plant system activities. - PS.03.01.05.c. Evaluate the impact of using genetically modified agricultural and ornamental crops on other production practices. - BS.03.04. Apply biotechnology principles, techniques, and processes to enhance plant and animal care and production (e.g., selective breeding, pharmaceuticals, biodiversity, etc.).
